# The Effect of Technology Use in Reducing Social Isolation or Loneliness in Older Adults: A Systematic Review

**DOI:** 10.1093/geroni/igaa057.3400

**Published:** 2020-12-16

**Authors:** Yoon Chung Kim, Gail Kohn, Carenado Davis, Pamela Saunders

**Affiliations:** 1 Georgetown University, Washington, District of Columbia, United States; 2 Age-Friendly DC, Washington, District of Columbia, United States; 3 Wake Technical Community College, Raleigh, North Carolina, United States

## Abstract

The US population over 65 is projected to increase to 21% by 2050. Given mobility issues arising from health concerns, economic status changes, loss of friends and partners, older people are at a higher risk for social isolation and loneliness. Since the declaration of national emergency for COVID-19 on March 13, 2020, many older adults have not been able to connect with others in traditional ways. Instead, activities and contacts have been facilitated virtually via videoconferencing or phone calls to maintain physical and social distance. Amidst COVID-19, the transition to using technologies to connect socially and reduce loneliness has been a critical factor in preventing social isolation and loneliness. Identifying effective strategies involving the use of technology, designing new ways to deliver services virtually, and developing educational programs to promote technology is vitally necessary. This systematic review explored the relationship between technology use and social isolation or loneliness, and examined interventions that reduced social isolation in older adults. A unified strategy was used to systematically search seven databases (MEDLINE, PubMed, Embase, CINAHL, Web of Science, PsycInfo, and AgeLine) to examine qualitative and quantitative studies published in English between 2010 and 2020. Preliminary results indicate that technology can alleviate social isolation and loneliness in older adults despite some mixed results. The findings of this study will provide a foundation for policymakers and practitioners to shape policies and design programs that help older adults to alleviate social isolation and loneliness, particularly amidst the COVID-19 pandemic.

